# Supplementary material for: Sweep Sampling Comparison of Terrestrial Insect Communities Associated with Herbaceous Stratum in the Riparian Zone of the Miho River, Korea
Source: Insects. 2022 May 25;13(6):497. doi: 10.3390/insects13060497 (PMC9224486; doi:10.3390/insects13060497)
Supplement: Supplementary file 1 [file insects-13-00497-s001.zip › insects-1724938-Supplementary.pdf]

Table S1. Matching information and rates of insect herbivore host plant and vascular plant flora of the sites

Terrestrial herbivorous insects	Host plants referred	*Chenopodium album* 20	*Beckmannia syzigachne* 2020	*Beckmannia syzigachne* 2021	*Artemisia indica* 2020	*Artemisia indica* 2021
Cardipennis sulcithorax (Hustache, 1916)	*Humulus japonicus* [44]	0	3	0	6	0
Psallus (*Calopsallus*) *clarus* Kerzhner, 1988	*Quercus dentata* [41]	0	0	0	1	0
Dryodurgades lamellaris Vilbaste, 1968	*Pteridium aquilinum* var. *latiusculum* and *Pueraria lobata* [59]	0	0	0	0	4
Tephritidae sp.	unknown	0	0	1	0	0
Metalimnus steini (Fieber, 1869)	unknown	0	0	1	0	0
Corythucha marmorata Uhler, 1878	polyphagy [55]	0	1	3	0	58
Ziczacella steggerdai (Ross, 1965)	*Humulus japonicus* [53]	0	11	3	0	0
Eucera (*Eucera*) *sociabilis* (Smith, 1873)	Assumed polyphagy	0	0	0	0	1
Stigmatonotum rufipes (Motschulsky, 1866)	Collected on *Hypericum japonicum* [62]	0	1	0	0	1
Macrosteles brunnescens Anufriev, 1968	unknown	0	0	0	0	10
Acanthocoris sordidus (Thunberg, 1783)	Solanaceae and Convolvulaceae [41]	0	0	0	0	2
Apidae sp.	assumed polyphagy	0	0	0	0	1
Agapanthia (*Epoptes*) *amurensis* Kraatz, 1879	Asteraceae [59]	0	0	0	0	1
Cassida nebulosa Linnaeus, 1758	*Chenopodium album*, *Chenopodium album* var. *centrorubrum* [42]	2	0	0	0	0
Goniagnathus (*Epitephra*) *rugulosus* (Haupt, 1917)	unknown	0	0	0	1	2
Polymerus (*Polymerus*) *amurensis* Kerzhner, 1988	unknown	0	5	0	0	0
Austroasca vittata (Lethierry, 1884)	Genus *Artemisia* [58]	0	0	0	776	625
Plagiognathus yomogi Miyamoto, 1969	*Artemisia indica* [41]	0	0	0	0	31
Pachygrontha antennata (Uhler, 1860)	Poaceae [41]	1	0	0	0	1
Orthezia urticae (Linnaeus, 1758)	Asteraceae [59]	0	0	0	29	0
Eysarcoris gibbosus Jakovlev, 1904	*Leonurus japonicus* [41]	0	0	1	0	0
Nakaharanus sagittarius Kwon and Lee, 1979	unknown	0	0	0	0	3
Galerucella (*Galerucella*) *grisescens* (Joannis, 1865)	*Persicaria thunbergii*, *Rumex crispus*, Genus *Persicaria* [42]	1	0	0	0	0

Cicadella viridis (Linnaeus, 1758)	Polyphagy [59]	0	0	0	0	1
Cicadellidae sp. 1	unknown	0	0	0	0	25
Cicadellidae sp. 2	unknown	0	0	0	0	2
Cicadellidae sp. 3	unknown	0	0	0	0	1
Cicadellidae sp. 4	unknown	0	0	3	0	0
Cicadellidae sp. 5	unknown	0	0	2	0	0
Piesma capitatum (Wolff, 1804)	Other *Piesma* species' host is Chenopodiaceae [53]	16	0	0	0	0
Orthotylus flavosparsus (Sahlberg, 1841)	Chenopodiaceae [41]	83	5	0	0	0
Dorytomus (*Dorytomus*) *imbecillus* Faust, 1883	Genus *Salix* [59]	0	17	29	0	0
Plagiognathus amurensis Reuter, 1883	Polyphagy [41]	23	28	1	18	0
Europiella artemisiae (Becker, 1864)	*Artemisia indica* [41]	0	1	0	207	419
Taylorilygus apicalis (Fieber, 1861)	Polyphagy but prefer Asteraceae [41]	0	0	0	0	12
Lema (*Lema*) *concinnipennis* Baly, 1865	*Commelina communis* [42]	0	1	0	0	0
Plagiodera versicolora (Laicharting, 1781)	*Salix koreensis* [42]	0	0	2	0	0
Chrysomela vigintipunctata vigintipunctata (Scopoli, 1763)	*Salix koreensis* [42]	0	0	1	0	0
Chrysomelidae sp.	unknown	0	0	3	0	17
Lissorhoptrus oryzophilus Kuschel, 1952	Poaceae, Cyperaceae [56]	0	1	0	0	1
Chlorops oryzae Matsumura, 1915	Poaceae [63]	0	10	8	0	45
Geotomus convexus Hsiao, 1977	Assumed polyphagy	0	0	1	0	0
Dorytomus (*Dorytomus*) *roelofsi* Faust, 1882	Genus *Salix* [45]	0	0	1	0	0
Rhopalus (*Aeschyntelus*) *maculatus* (Fieber, 1837)	Polyphagy [41]	0	0	0	1	0
Fleutiauxia armata (Baly, 1874)	*Cudrania tricuspidata* [42]	0	1	0	0	0
Cletus punctiger (Dallas, 1852)	Polyphagy [41]	1	0	0	0	0
Chrysolina (*Anopachys*) *aurichalcea* (Mannerheim, 1825)	*Artemisia indica* [42]	0	0	0	0	1
Cosmobaris scolopacea (Germar, 1819)	Chenopodiaceae [51]	0	0	0	0	1
Crepidodera plutus (Latreille, 1804)	Genus *Salix* [43]	0	1	1	0	0
Hypera (*Hypera*) *postica* (Gyllenhal, 1813)	Fabaceae [50]	0	0	0	1	0
Orthocephalus funestus Jakovlev, 1881	*Artemisia indica* [41]	0	0	0	3	10

Nysius hidakai Nakatani, 2015	Other *Nysius species* usually use Asteraceae as a host [41]	3	0	0	51	0
Cassida piperata Hope, 1842	*Achyranthes bidentata* var. *japonica*, *Chenopodium album* var. *centrorubrum*, *Amaranthus lividus*, *Commelina communis* [43]	2	0	0	0	0
Apolygus spinolae (Meyer-Dür, 1841)	Polyphagy [41]	0	0	0	7	2
Apis mellifera Linnaeus, 1758	Assumed polyphagy	0	0	0	0	2
Dimorphopterus japonicus (Hidaka, 1959)	*Miscanthus sinensis* var. purpurascens, *Phragmites japonica* [41]	0	0	0	0	1
Deraeocoris (*Camptobrochis*) *pulchellus* (Reuter, 1906)	*Artemisia indica* and *Erigeron annuus* ([41]	0	0	0	3	2
Ceutorhynchus (*Ceutorhynchus*) *albosuturalis* (Roelofs, 1875)	Brassicaceae [57, 44]	68	1	0	0	0
Heteraspis lewisii Baly, 1874	*Ampelopsis brevipedunculata*, *Vitis coignetiae*, *Parthenocissus tricuspidata* [43]	0	0	0	0	3
Tenthredinidae sp.	unknown	0	0	1	0	0
Gastrophysa atrocyanea Motschulsky, 1860	*Rumex crispus* and *Rumex japonicus* [12]	0	1	0	0	1
Luprops orientalis (Motschulsky, 1868)	Other genus *Luprops* species exhibit polyphagy [64]	0	0	1	0	0
Capsus cinctus (Kolenati, 1845)	Recorded hosts are *Eleusine indica* and Genus *Calamagrostis* so Poaceae assumed as a host [54, 60]	0	107	188	0	1
Apionidae sp.	unknown	0	0	1	0	0
Apolygus lucorum (Meyer-Dür, 1843)	Polyphagy but generally found on *Artemisia indica* [61, 41]	1	0	0	32	48
Ceutorhynchus (*Ceutorhynchus*) *filiae* Dalla Torre, 1922	Brassicaceae [57, 44]	0	0	0	2	0
Metasalis populi (Takeya, 1932)	Genus *poplar*, *Platanus*, *Salix*, *Populus* [41]	0	3	0	0	0
Nezara antennata Scott, 1874	Polyphagy but prefer Fabaceae [41]	0	1	0	0	0
Psylliodes (*Psylliodes*) *attenuata* (Koch, 1803)	*Humulus japonicus* [48]	199	257	0	8	0
Stenodema calcarata (Fallén, 1807)	Poaceae and Cyperaceae [41]	0	3	1	0	0
Psilarthroides czerskyi (Zaslavskij, 1956)	*Humulus japonicus* [44]	0	6	0	0	3
Cardipennis shaowuensis (Voss, 1958)	*Humulus japonicus* [44]	2	6	0	0	0
	**Matching rate**	**100**	**82.6**	**66.7**	**87.5**	**66.7**

Table S2. Vascular plant flora of Miho river by each plant species community

Plant species	2020 *C. album*	2020 *B. syzigachne*	2021 *B. syzigachne*	2020 *A. indica*	2021 *A. indica*
Equisetum arvense L.		1	1	1	1
Salix koreensis Andersson		1	1		
Morus alba L.					1
Humulus japonicus Sieboid & Zucc.	1	1	1		1
Persicaria lapathifolia (L.) Gray	1				
Rumex crispus L.		1	1	1	1
Arenaria serpyllifolia L.					1
Stellaria alsine var. *undulata* (Thunb.) Ohwi	1				
Humulus japonicus Sieboid & Zucc.	1	1	1		1
Persicaria lapathifolia (L.) Gray	1				
Rumex crispus L.		1	1	1	1
Arenaria serpyllifolia L.					1
Stellaria alsine var. *undulata* (Thunb.) Ohwi	1				
Stellaria aquatica (L.) Scop.			1		
Stellaria media (L.) Vill.		1			
Chenopodium album L.	1		1		1
Chenopodium album var. *centrorubrum* Makino		1		1	
Chenopodium bryoniaefolium Bunge	1				
Chenopodium ficifolium Smith	1				
Chenopodium glaucum L.	1				
Ranunculus sceleratus L.	1				
Menispermum dauricum DC.			1		
Chelidonium majus var. *asiaticum* (Hara) Ohwi		1	1	1	1
Papaver rhoeas L.				1	
Barbarea orthoceras Ledeb.	1				
Brassica napus L.	1				
Capsella bursapastoris (L.) L.W.Medicus	1	1			
Cardamine flexuosa With.	1				
Descurainia pinnata Britton	1				
Descurainia sophia (L.) Webb ex Prantl	1				
Draba nemorosa L.	1				
Lepidium sativum L.				1	
Lepidium virginicum L.				1	
Rorippa palustris (Leyss.) Besser	1		1		
Thlaspi arvense L.	1	1	1		
Potentilla supina L.	1				
Vicia amoena Fisch. ex DC.				1	
Vicia angustifolia var. *segetilis* (Thuill.) K.Koch.				1	1
Vicia villosa Roth					1

Oenothera biennis L.				1	1
Anthriscus sylvestris (L.) Hoffm.			1		
Anthriscus sylvestris var. *hirtifructus* (Ohwi) Hara		1			
Oenanthe javanica (Blume) DC.			1		
Galium spurium var. *echinospermon* (Wallr.) Hayek		1	1		1
Trigonotis peduncularis (Trevir.) Benth. ex Hemsl.	1		1		
Lamium amplexicaule L.	1				
Leonurus japonicus Houtt.		1			
Scutellaria indica L.				1	
Mazus pumilus (Burm.f.) Steenis			1		
Veronica arvensis L.	1				
Veronica peregrina L.	1				
Veronica undulata Wall.	1				
Ambrosia artemisiifolia L.	1				
Artemisia indica Willd.		1		1	1
Artemisia selengensis Turcz. ex Besser			1		
Coreopsis lanceolata L.					1
Erigeron annuus (L.) Pers.		1	1		1
Hemistepta lyrata Bunge			1	1	1
Ixeris polycephala Cass.				1	1
Taraxacum officinale Weber				1	
Youngia japonica (L.) DC.			1		
Agrostis dimorpholemma Ohwi					1
Alopecurus aequalis Sobol.	1		1		
Beckmannia syzigachne (Steud.) Fernald		1	1		
Bromus japonicus Thunb.			1		1
Miscanthus sinensis var. *purpurascens* (Andersson) Rendle		1			
Phragmites communis Trin.		1			
Phragmites japonica Steud.	1				1
Poa pratensis L.		1			
Carex aphanolepis Franch. & Sav.				1	
Carex breviculmis R.Br.		1			
Carex glabrescens Ohwi		1		1	
Carex miyabei Franch.		1		1	
Stellaria aquatica (L.) Scop.			1		
Stellaria media (L.) Vill.		1			
Chenopodium album L.	1		1		1
Chenopodium album var. *centrorubrum* Makino		1		1	
Chenopodium bryoniaefolium Bunge	1				
Chenopodium ficifolium Smith	1				
Chenopodium glaucum L.	1				
Ranunculus sceleratus L.	1				

Menispermum dauricum DC.			1		
Chelidonium majus var. *asiaticum* (Hara) Ohwi		1	1	1	1
Papaver rhoeas L.				1	
Barbarea orthoceras Ledeb.	1				
Brassica napus L.	1				
Capsella bursapastoris (L.) L.W.Medicus	1	1			
Cardamine flexuosa With.	1				
Descurainia pinnata Britton	1				
Descurainia sophia (L.) Webb ex Prantl	1				
Draba nemorosa L.	1				
Lepidium sativum L.				1	
Lepidium virginicum L.				1	
Rorippa palustris (Leyss.) Besser	1		1		
Thlaspi arvense L.	1	1	1		
Potentilla supina L.	1				
Vicia amoena Fisch. ex DC.				1	
Vicia angustifolia var. *segetilis* (Thuill.) K.Koch.				1	1
Vicia villosa Roth					1
Oenothera biennis L.				1	1
Anthriscus sylvestris (L.) Hoffm.			1		
Anthriscus sylvestris var. *hirtifructus* (Ohwi) Hara		1			
Oenanthe javanica (Blume) DC.			1		
Galium spurium var. *echinospermon* (Wallr.) Hayek		1	1		1
Trigonotis peduncularis (Trevir.) Benth. ex Hemsl.	1		1		
Lamium amplexicaule L.	1				
Leonurus japonicus Houtt.		1			
Scutellaria indica L.				1	
Mazus pumilus (Burm.f.) Steenis			1		
Veronica arvensis L.	1				
Veronica peregrina L.	1				
Veronica undulata Wall.	1				
Ambrosia artemisiifolia L.	1				
Artemisia indica Willd.		1		1	1
Artemisia selengensis Turcz. ex Besser			1		
Coreopsis lanceolata L.					1
Erigeron annuus (L.) Pers.		1	1		1
Hemistepta lyrata Bunge			1	1	1
Ixeris polycephala Cass.				1	1
Taraxacum officinale Weber				1	
Youngia japonica (L.) DC.			1		
Agrostis dimorpholemma Ohwi					1
Alopecurus aequalis Sobol.	1		1		
Beckmannia syzigachne (Steud.) Fernald		1	1		

Bromus japonicus Thunb.			1		1
Miscanthus sinensis var. *purpurascens* (Andersson) Rendle		1			
Phragmites communis Trin.		1			
Phragmites japonica Steud.	1				1
Poa pratensis L.		1			
Carex aphanolepis Franch. & Sav.				1	
Carex breviculmis R.Br.		1			
Carex glabrescens Ohwi		1		1	
Carex miyabei Franch.		1		1	
Carex neurocarpa Maxim.					1

Table S3. Insect fauna of Miho river by each plant species community

Insect species	2020 *C. album*	2020 *B. syzigachne*	2021 *B. syzigachne*	2020 *A. indica*	2021 *A. indica*
Stricticollis valgipes (Marseul, 1875)					6
Apionidae sp.			1		
Cantharis (*Cyrtomoptila*) *plagiata* Heyden, 1889	5	38	7	20	35
Chlaenius (*Lissauchenius*) *naeviger* Morawitz, 1862					1
Agapanthia (*Epoptes*) *amurensis* Kraatz, 1879					1
Cassida nebulosa Linnaeus, 1758	2				
Cassida piperata Hope, 1842	2				
Chrysolina (*Anopachys*) *aurichalcea* (Mannerheim, 1825)					1
Chrysomela vigintipunctata (Scopoli, 1763)			1		
Chrysomelidae sp.			3		17
Crepidodera plutus (Latreille, 1804)		1	1		
Fleutiauxia armata (Baly, 1874)		1			
Galerucella (*Galerucella*) *grisescens* (Joannis, 1865)	1				
Gastrophysa atrocyanea Motschulsky, 1860		1			1
Heteraspis lewisii Baly, 1874					3
Lema (*Lema*) *concinnipennis* Baly, 1865		1			
Plagiodera versicolora (Laicharting, 1781)			2		
Psylliodes (*Psylliodes*) *attenuata* (Koch, 1803)	199	257		8	
Calvia muiri (Timberlake, 1943)			2		
Harmonia axyridis (Pallas, 1773)				1	
Hippodamia (*Hemisphaerica*) *tredecimpunctata* (Linnaeus, 1758)	1				
Nephus (*Bipunctus*) *incinctus* (Mulsant, 1850)					1
Propylea japonica (Thunberg, 1781)	6	6	7	35	27
Scymnus (*Neopullus*) *babai* Sasaji, 1971			5		
Scymnus (*Pullus*) *posticalis* Scard, 1912			1		1
Cryptophagidae sp.					1
Cardipennis shaowuensis (Voss, 1958)	2	6			

Cardipennis sulcithorax (Hustache, 1916)		3		6	
Ceutorhynchus (*Ceutorhynchus*) *albosuturalis* (Roelofs, 1875)	68	1			
Ceutorhynchus (*Ceutorhynchus*) *filiae* Dalla Torre, 1922				2	
Cosmobaris scolopacea (Germar, 1819)					1
Dorytomus (*Dorytomus*) *imbecillus* Faust, 1883		17	29		
Dorytomus (*Dorytomus*) *roelofsi* Faust, 1882			1		
Hypera (*Hypera*) *postica* (Gyllenhal, 1813)				1	
Psilarthroides czerskyi (Zaslavskij, 1956)		6			3
Elateridae sp.					1
Lissorhoptrus oryzophilus Kuschel, 1952		1			1
Lathridiidae sp. 1			1		
Lathridiidae sp. 2					2
Malachius (*Malachius*) *prolongatus* Motschulsky, 1866	2	1		12	4
Mordellidae sp.					3
Psammoecus sp.					5
Silvanidae sp.					2
Paederus (*Heteropaederus*) *fuscipes* Curtis, 1826	1				
Luprops orientalis (Motschulsky, 1868)			1		
Asilidae sp.					1
Chlorops oryzae Matsumura, 1915		10	8		45
Dolichopus nitidus Fallen, 1823			3	26	219
Sciasminettia similis Shatalkin, 2000					1
Colobaea sp.					1
Sepedon aenescens Wiedemann, 1830		1			
Sphaerophoria menthastri (Linnaeus, 1758)			2		
Tephritidae sp.			1		
Amphiareus obscuriceps (Poppius, 1909)					1
Anthocoridae sp.					92
Orius (*Heterorius*) *sauteri* (Poppius, 1909)				31	
Metatropis tesongsanicus Josifov, 1975		1			
Austroasca vittata (Lethierry, 1884)				776	625
Cicadella viridis (Linnaeus, 1758)					1
Cicadellidae sp. 1					25
Cicadellidae sp. 2					2
Cicadellidae sp. 3					1
Cicadellidae sp. 4			3		
Cicadellidae sp. 5			2		
Dryodurgades lamellaris Vilbaste, 1968					4
Goniagnathus (*Epitephra*) *rugulosus* (Haupt, 1917)				1	2
Macrosteles brunnescens Anufriev, 1968					10
Metalimnus steini (Fieber, 1869)			1		
Nakaharanus sagittarius Kwon and Lee, 1979					3
Ziczacella steggerdai (Ross, 1965)		11	3		

Acanthocoris sordidus (Thunberg, 1783)					2
Cletus punctiger (Dallas, 1852)	1				
Geotomus pygmaeus (Dallas, 1851)			1		
Dimorphopterus japonicus (Hidaka, 1959)					1
Geocoris (*Piocoris*) *varius* (Uhler, 1860)					1
Nysius plebejus Distant, 1883	3			51	
Pachygrontha antennata (Uhler, 1860)	1				1
Stigmatonotum rufipes (Motschulsky, 1866)		1			1
Apolygus lucorum (Meyer-Dür, 1843)	1			32	48
Apolygus spinolae (Meyer-Dür, 1841)				7	2
Capsus cinctus (Kolenati, 1845)		107	188		1
Deraeocoris (*Camptobrochis*) *pulchellus* (Reuter, 1906)				3	2
Europiella artemisiae (Becker, 1864)		1		207	419
Orthocephalus funestus Jakovlev, 1881				3	10
Orthotylus flavosparsus (Sahlberg, 1841)	83	5			
Plagiognathus amurensis Reuter, 1883	23	28	1	18	
Plagiognathus yomogi Miyamoto, 1969					31
Polymerus (*Polymerus*) *amurensis* Kerzhner, 1988		5			
Psallus (*Calopsallus*) *clarus* Kerzhner, 1988				1	
Stenodema calcarata (Fallén, 1807)		3	1		
Taylorilygus apicalis (Fieber, 1861)					12
Orthezia urticae (Linnaeus, 1758)				29	
Eysarcoris gibbosus Jakovlev, 1904			1		
Nezara antennata Scott, 1874		1			
Zicrona caerulea (Linnaeus, 1758)					3
Piesma capitatum (Wolff, 1804)	16				
Rhopalus (*Aeschyntelus*) *maculatus* (Fieber, 1837)				1	
Corythucha marmorata Uhler, 1878		1	3		58
Metasalis populi (Takeya, 1932)		3			
Apidae sp.					1
Apis mellifera Linnaeus, 1758					2
Eucera (*Eucera*) *sociabilis* (Smith, 1873)					1
Argidae sp.			1		
Nylanderia flavipes (Smith, 1874)					1
Nylanderia sakurae (Ito, 1914)					1
Tetramorium tsushimae Emery, 1925				71	177
Ichneumonidae sp.			3		2
Polistes snelleni Saussure, 1862				1	
Paracercion calamorum (Ris, 1916)		2			

Table S4. Comparison table of the Sørensen similarity indices for the terrestrial insects in each plant community with sampling units from 2020

Sørensen similarity		*C. album*			*B. syzigachne*			*A. indica*		
		a	b	ab	a	b	ab	a	b	ab
C. album	a									
	b	**0.62**								
	ab	**0.71**	**0.94**							
B. syzigachne	a	0.30	0.36	0.34						
	b	0.28	0.34	0.32	**0.62**					
	ab	0.31	0.36	0.34	**0.88**	**0.79**				
A. indica	a	0.21	0.35	0.33	0.20	0.27	0.26			
	b	0.25	0.37	0.35	0.22	0.29	0.27	**0.80**		
	ab	0.24	0.35	0.33	0.21	0.28	0.26	**0.86**	**0.96**	

*a, initial 50 sweeps over 25 m; b, final 50 sweeps over 25 m; ab, 100 sweep total over 50 m. *Values above 0.5 are provided in bold.

Table S5. Comparison table of the Bray–Curtis similarity indices for the terrestrial insects among the plant communities with sampling units from 2020

Bray–Curtis similarity		*C. album*			*B. syzigachne*			*A. indica*		
		a	b	ab	a	b	ab	a	b	ab
C. album	a									
	b	**0.44**								
	ab	**0.56**	**0.76**							
B. syzigachne	a	**0.37**	**0.46**	**0.53**						
	b	**0.54**	**0.52**	**0.38**	**0.58**					
	ab	**0.29**	**0.39**	**0.52**	**0.80**	**0.51**				
A. indica	a	0.02	0.03	0.04	0.04	0.04	0.04			
	b	0.04	0.09	0.08	0.06	0.10	0.08	**0.64**		
	ab	0.02	0.05	0.05	0.05	0.06	0.06	**0.77**	**0.55**	

*a, initial 50 sweeps over 25 m; b, final 50 sweeps over 25 m; ab, total of 100 sweeps over 50 m. *Values above 0.1 are in bold.

Table S6. Comparison table of the Sørensen similarity indices for the terrestrial insects among the plant communities and years

Sørensen similarity	*B. syzigachne* 2020	*B. syzigachne* 2021	*A. indica* 2020	*A. indica* 2021
B. syzigachne 2020				
B. syzigachne 2021	0.34			
A. indica 2020	0.26	0.15		
A. indica 2021	0.25	0.2	0.29	

Table S7. Comparison table of the Bray–Curtis similarity indices for the terrestrial insects among the plant communities and years

Bray–Curtis similarity	*B. syzigachne* 2020	*B. syzigachne* 2021	*A. indica* 2020	*A. indica* 2021
B. syzigachne 2020				
B. syzigachne 2021	**0.38**			
A. indica 2020	0.06	0.02		
A. indica 2021	0.05	0.03	**0.62**	

*Values above 0.1 are highlighted in bold.

Table S8. The number of species by the sampling in 2020

	C. album		*B. syzigachne*		*A. indica*	
**Sample tag**	a	b	a	b	a	b
**Number of species**	10	16	23	19	18	22
**Number of individuals**	164	253	344	176	832	511

*a : initial 50 sweeps over 25 m. b: final 50 sweeps over 25 m.

Table S9. The number of species by the sampling in 2021

Plant community and sampling unit order number	Number of species	Number of individuals
***B. syzigachne* 1**	8	25
***B. syzigachne* 2**	6	15
***B. syzigachne* 3**	5	28
***B. syzigachne* 4**	9	23
***B. syzigachne* 5**	5	16
***B. syzigachne* 6**	5	23
***B. syzigachne* 7**	11	47
***B. syzigachne* 8**	5	31
***B. syzigachne* 9**	11	47
***B. syzigachne* 10**	10	29
***A. indica* 1**	24	253
***A. indica* 2**	24	358
***A. indica* 3**	16	166
***A. indica* 4**	22	199
***A. indica* 5**	23	162
***A. indica* 6**	22	123
***A. indica* 7**	22	154
***A. indica* 8**	17	221
***A. indica* 9**	23	178
***A. indica* 10**	16	113
